# Towards improving diagnosis of memory loss in general practice: TIMeLi diagnostic test accuracy study protocol

**DOI:** 10.1186/s12875-016-0475-2

**Published:** 2016-07-19

**Authors:** Sam T. Creavin, Sarah J. Cullum, Judy Haworth, Lesley Wye, Antony Bayer, Mark Fish, Sarah Purdy, Yoav Ben-Shlomo

**Affiliations:** School of Social and Community Medicine, University of Bristol, 39 Whatley Road, Bristol, BS8 2PS UK; North Bristol NHS Trust Southmead Hospital, Southmead Road, Westbury-on-Trym, Bristol, BS10 5NB UK; Division of Population Medicine, Cardiff University School of Medicine University Hospital Llandough, Penarth, CF64 2XX UK; Department of Neurology, Musgrove Park Hospital, Taunton, Somerset TA1 5DA UK

**Keywords:** Dementia, Primary care, General practice, Sensitivity and specificity, Diagnostic tests

## Abstract

**Background:**

People with cognitive problems, and their families, report distress and uncertainty whilst undergoing evaluation for dementia and perceive that traditional diagnostic evaluation in secondary care is insufficiently patient centred. The James Lind Alliance has prioritised research to investigate the role of primary care in supporting a more effective diagnostic pathway, and the topic is also of interest to health commissioners. However, there are very few studies that investigate the accuracy of diagnostic tests for dementia in primary care.

**Methods:**

We will conduct a prospective diagnostic test accuracy study to evaluate the accuracy of a range of simple tests for diagnosing all-cause-dementia in symptomatic people aged over 70 years who have consulted with their general practitioner (GP). We will invite eligible people to attend a research clinic where they will undergo a range of index tests that a GP could perform in the surgery and also be assessed by a specialist in memory disorders at the same appointment. Participating GPs will request neuroimaging and blood tests and otherwise manage patients in line with their usual clinical practice. The reference standard will be the consensus judgement of three experts (neurologist, psychiatrist and geriatrician) based on information from the specialist assessment, GP records and investigations, but not including items in the index test battery. The target condition will be all-cause dementia but we will also investigate diagnostic accuracy for sub-types where possible. We will use qualitative interviews with patients and focus groups with clinicians to help us understand the acceptability and feasibility of diagnosing dementia in primary care using the tests that we are investigating.

**Discussion:**

Our results will help clinicians decide on which tests to perform in someone where there is concern about possible dementia and inform commissioning of diagnostic pathways.

## Background

Dementia is a syndrome of global cognitive impairment which represents a decline from a previous level of functioning, often with behavioural and psychiatric symptoms [1], that affects around 750,000 people in the UK, of whom half have a diagnosis recorded on GP records [2]. Dementia, recently termed “major neurocognitive disorder” [3, 4], is categorised according to clinical features and presumed aetiology with the common clinical diagnoses being Alzheimer’s [5, 6], ischaemic cerebrovascular disease [7], Lewy body disease [8, 9], tauopathy/frontotemporal dementia [10], and other rarer causes. All-cause-dementia is also defined, without additional specified clinical features needed for the subtype definitions [11–13].

In the population, Alzheimer’s disease and vascular pathology are the major neuropathological features that are associated with dementia syndrome [14], but there are often multiple contributing elements [14, 15]. The association between Alzheimer’s disease pathology and dementia is strongest in the young-old and weakens with age [16], leading some investigators to question the value of presumed aetiological diagnosis in a population where mixed pathology is usual [17, 18].

Mild cognitive impairment (MCI) [19] is a syndrome of cognitive impairment that is greater than expected when accounting for age and educational attainment but that does not affect activities of daily life. MCI affects between 0.1 and 42 % of adults depending on which definition is used [20], and the prognosis in general practice is variable: approximately 25 % of people develop dementia within three years but around 40 % revert to normal [21]. Experience in clinical general practice is that when there are concerns about impaired cognition these are focussed primarily on the possibility of dementia rather than MCI, but inevitably some people who are evaluated for possible dementia will be diagnosed with MCI. In this protocol we include people who are ultimately diagnosed as having MCI when we refer to a person consulting with a GP about possible dementia (e.g. under “participants”), because it would be unusual for a person to consult a GP about possible MCI.

People with cognitive problems and their families experience uncertainty while undergoing evaluation for possible dementia [22] and the role of primary care in supporting a more effective route to diagnosis has been identified as a priority for health research [23]. Health policy changed significantly between 2010-2015: in the USA Medicare has included an annual cognitive check-up since 2013 [24]; in England case-finding for dementia started in 2014 [25] and more recently GPs have been encouraged to take a more active role in diagnosing dementia independent of a specialist opinion [26].

Despite this change in health policy, few research studies exist to provide an evidence-based approach to the diagnosis of dementia in general practice and by GPs [27–33]. Often tests have been evaluated as tools for screening rather than diagnosis [34] and commonly used tests, such as the Montreal Cognitive Assessment (MOCA [35]) and Informant Questionnaire for Cognitive disorders in the Elderly (IQCODE [36]) have not been well evaluated in primary care [37, 38]. GPs are uncertain about diagnosing dementia and report working collaboratively with the memory nurse over up to four consultations before reaching a diagnosis [39].

We previously found that the diagnostic accuracy of simple questions concerning functioning and independent living was comparable to longer more established measures of cognitive functioning in a group of men who had been screened for cognitive problems [40]. Here we describe a prospective study to evaluate the accuracy of a range of tests for diagnosing dementia in a primary care setting.

## Methods

### Summary

We will conduct a prospective diagnostic test cohort study to evaluate the accuracy of a range of index tests (detailed below) for diagnosing dementia in symptomatic adults over the age of 70 years. The primary target condition is all-cause dementia; we will also examine the diagnostic accuracy for probable Alzheimer’s disease as compared to other causes of dementia. This study has been peer reviewed by the funding bodies. The background, aims and broad methods were reviewed as part of a competitive fellowship award. The funders were fully aware that over the course of the fellowship a more detailed protocol would be produced and there may be minor changes to the design in light of further work. These amendments are not re-reviewed by the funder.

### Participants

#### Setting

We will conduct the study in GP practices in the Bristol, North Somerset and South Gloucestershire region, in the South West of England with a total population of 972,417 people, with 16 % aged over 65 years and 11 % aged over 70 years. All 54 practices in the NIHR Western Clinical Research Network (CRN) of GP practices contributing to NHS (National Health Service) research infrastructure ([41]) will be invited to take part and refer patients to the study team. Research clinics will be held in practices within the CRN selected on the basis of geographic accessibility for local participants.

#### Recruitment

We will include people aged 70 years and over (i.e. who have had their 70^th^ birthday) where concerns have been raised in the community about the possibility of dementia by the patient or others (including GP), but in whom the diagnosis has yet to be confirmed. The symptoms must have been present for at least six months and been gradual in onset and progression. We will exclude people with clinical “red-flags”: co-incident tremor, weakness or dysphasia; existing diseases listed in Table 1; mental health problems needing secondary care input; terminal illness; or inability to attend research clinic with an informant. People with these conditions will almost always need specialist input to make a diagnosis of dementia, and an informant history is required for a robust diagnosis of dementia. Additionally we will exclude people with severe dementia (operationalised as lack of capacity to consent) as diagnosis in this group is less challenging.

**Table 1:** Conditions resulting in exclusion from TIMeLi study

Prior diagnosis of a parkinsonian condition (including Parkinson’s disease)	
Multiple sclerosis	
Learning disability	
Motor neuron disease	
Huntington’s disease	
Registered blind	
Severe hearing impairment (operationalised as unable to use telephone)	

**Fig. 1:**
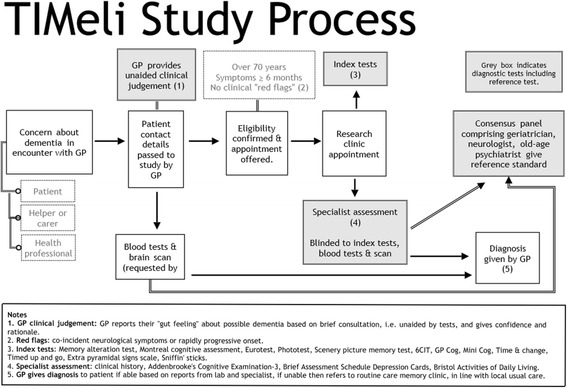
TIMeLi study process

Figure 1 outlines the process of the study. GPs will pass details on potential participants, after participant consent, to the research study by completing a template form that is then emailed to a secure nhs.net email address [42]. At the time of referral, the GPs are asked to state their prior belief (“gut feeling”) concerning the diagnosis and their confidence in this diagnosis (see “test methods | index tests” below for more detail). No prior testing is required to determine eligibility for the study, the only requirements are listed in the inclusion and exclusion criteria above. Investigations such as blood tests and neuroimaging will be requested by the GP in line with their usual clinical practice and can be conducted in parallel with attendance at the study clinic as the results will not be available to the researcher at the clinic. An administrator will process forms from the GP so that the researcher’s clinical evaluation of participants is blinded to the GP judgement, and this will only be made available to the study team at the analysis stage.

#### Sampling

The eligible study population will be defined by a consecutive series of patients who meet the criteria for recruitment, but we recognise that not everybody who is eligible will participate. Table 2 shows some of the reasons for non-participation of eligible patients and how we will try to minimise bias.

**Table 2:** Possible reasons for non-participation of eligible patients

Potential barrier to recruitment	How we will address this
GP factors	
Not thinking of the study when it is relevant	Computer prompts when relevant problem code entered
Being too busy to discuss it with patients	Computer prompt to record this
Believing that a patient is not suitable	Computer prompt to record this
Patient factors	
Difficulty accessing research clinic [day, time, travel]	Provide transport if needed, range of clinics on different days and times
Other health issues	Allow people to rearrange appointment if needed
Wanting time to decide	Allow people time to think and call back
No clear reason but declined	Computer prompt to record study declined

**Fig. 2:**
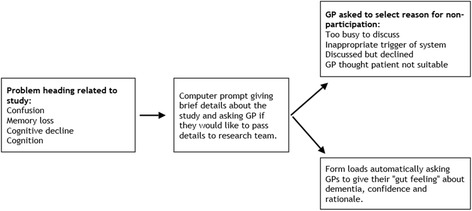
Computer prompts to aid participation

A particular problem is that GPs might not mention the study to people who are eligible because they forget to do so. To address this we will use an electronic prompt within the electronic medical record. Figure 2 outlines the computer prompts that are triggered when the GP enters a problem heading related to memory problems or cognitive difficulties during the consultation with the patient.

The computer prompts will help us to monitor practices for potentially eligible patients who have not participated using electronic searches of coded data in the electronic medical record.

#### Data collection

Research clinics will be held in GP practices in the NIHR Western CRN. At the research clinic one GP research doctor (STC) will see one patient-informant dyad for the index tests and simultaneously a dementia specialist doctor (JH) will see a different participant dyad for the specialist assessment of up to one hour in a separate room. Participants will have a 10-min rest before crossing over to see the other doctor and will be at the clinic for approximately 2.5 h in total. We aim that half of participants will see each doctor first, but the order of consultations will be determined by patient availability for appointments. So that the index tests and specialist assessment are conducted independently of investigations, test results will be electronically extracted by the study team at the end of the study, and will not be available on the day of the research clinic, though they will be available to inform the reference standard. We planned data collection and analysis in advance.

### Test methods

#### Index tests

GPs often use heuristics when making diagnoses [43–45], including the possibility of dementia [46, 47]. Referring GPs will be asked to give their “gut feeling” about the possibility of dementia, based on their brief interaction with the patient during the consultation at the time of referral. We will ask GPs to report whether they think the person has dementia, cognitive impairment (but not dementia), or is cognitively normal. In addition they are asked to rate their diagnostic confidence using a 10 cm visual analogue scale which is then converted to a percentage (prior probability) and their rationale for their opinion.

In deciding what simple index tests should be evaluated, we reviewed the literature to identify cognitive tests and also referred to a guide to clinicians about tests that could be used in primary care [48]. We selected tests for the index battery on the basis of the following criteria:

1. Available to use for free – i.e. not copyright (therefore MMSE [49] excluded);
2. Previously evaluated in a primary care setting in at least one study;
3. Not been evaluated in primary care before but conceptually of interest (Timed up and go; Sniffin’ sticks).

Based on these criteria we selected studies with good diagnostic accuracy (Youden index [50] of greater than 0.75 or a sensitivity or specificity of greater than 0.85 at the optimal reported threshold) and judged the studies diagnostic accuracy against the time taken to conduct the test, favouring brief tests with high specificity. On this basis the following tests (“index battery”) were selected to be included in the index battery: Memory alteration test (M@T, [51]), Eurotest [27], Phototest [52], Scenery picture memory test [53], 6CIT [54], GPCOG [55], Mini-Cog [56], Time and change [57], Timed up-and-go [58], Extra pyramidal signs scale [59], Sniffin’ sticks [60, 61]. We also included assessments of activities of daily living; the Pfeffer [62], Lawton [63] Katz [64], AD8 [65] and Informant questionnaire for cognitive disorders in the elderly (IQCODE) short version [36]. A different group of investigators have reviewed the use of cognitive tests in primary care [66], compared to the tests identified by that group our battery does not include memory impairment screen (MIS) [67] or abbreviated mental test (AMT) [68] but does include indicators which reflect similar aspects of cognitive testing and are possibly more culturally fair, and which have fewer restrictions on use. In the MIS (all rights reserved), patients are asked to read from a list of four words, then engaged in a distractor activity and finally scored on free and cued recall; in comparison Phototest (which is available for use under a creative commons license [69]) requires participants to identify six photos, perform a distractor task and then tests free and cued recall. We include six of the 10 items in the AMT in our index battery and exclude age and recognition of two people (which are arguably less discriminative in people without severe impairment) year of First World War and name of present monarch (which are arguably more culturally determined).

We did not initially include the Montreal Cognitive Assessment (MoCA, [35]) in the index battery as it was originally designed to diagnose or identify MCI, had been advocated for use in secondary care [48] and had not been investigated in primary care [38]. However, we revised our protocol in light of subsequent policy changes in 2015 that encouraged GPs to diagnose dementia in typical situations without referring to a specialist [26] using the MoCA as the preferred instrument. We replaced the M@T with the MoCA because we judged that including both the MoCA and the M@T would be overly burdensome for participants and have little added value.

Index tests will be performed as instructed by the original authors by a single doctor who has completed postgraduate training in general practice (STC), who will not be aware of any other clinical information about the participants, including the GPs “gut feeling” about the possibly of dementia. The full index battery takes around 25 min in a healthy person and around 50 min in a person with dementia.

#### Specialist assessment

A single dementia specialist (JH) will perform a standardised clinical evaluation lasting approximately an hour, comprising clinical history, the Addenbrooke’s Cognitive Examination third edition (ACE-III) [70], Brief Assessment Schedule Depression Cards (BASDEC) [71] and the Bristol activities of daily living questionnaire (BADL) [72]. The specialist will not have access to any investigation results because we want to assess the accuracy of clinical assessment by a specialist. If the specialist considers that further investigations and assessment are needed to exclude a rare dementia aetiology we will suggest to the referring GP that they may wish to refer the patient to the National Health Service (NHS) memory clinic. The specialist will be asked to reach a clinical judgement about the cognitive status of participants operationalised as normal, cognitive impairment, or dementia, as well as the most likely aetiology of the dementia based on the information available to them.

#### Reference standard

The reference standard will be the consensus judgement by an expert panel about the diagnosis of dementia, using information from the specialist assessment, blood tests, neuroimaging, and medical records (where needed). Information from the index battery and GP “gut feeling” will not contribute to the reference standard. We will allow the reference panel access to the results of any tests that have been conducted up to six months after a research clinic because in some cases special tests such as regional cerebral blood flow single photon emission computed tomography (SPECT) or dopamine transporter imaging (DAT) scans may have been requested and would help refine the reference standard. We will use a stepwise-reveal process for all items that contribute to the reference panel, starting with the anonymised demographics, medical history and clinical assessment from the research clinic, followed by blood tests and routine neuroimaging such as plain computed tomogram (CT) or plain magnetic resonance imaging (MRI), followed by additional information from medical records and additional tests such as DAT or SPECT scans or neuropsychology results (if available). We intend to use a staged decision making approach for assigning the final diagnosis [73] where each expert initially assigns a diagnosis independently and then discordant cases are discussed.

#### Definitions

To account for differences between definitions [74] we will apply three different criteria for dementia: Diagnostic and Statistical Manual of Mental Disorders (DSM) IV [12], DSM 5 [3] and the International Classification of Diseases Tenth edition (ICD 10 [11]). For cognitive impairment that does not meet criteria for dementia we will use two definitions: DSM 5 [3] mild neurocognitive disorder and Peterson MCI [19]. The final reference standard for the main analysis will be the consensus judgement about the presence or absence of dementia, cognitive impairment or normal cognition, based on the application of the three definitions above. An example of how we might assign diagnoses is in Table 3.

**Table 3:** Example of process for assigning the reference standard

Assessor	AB	MF	SJC
Role	Consultant geriatrician with interest in memory disorders	Consultant neurologist	Consultant old age psychiatrist
Study ID	XX1	XX1	XX1
Status: Dementia/major neurocognitive disorder DSM 5 or MCI [19]/mild neurocognitive disorder DSM 5 or Normal			
DSM^a^ IV [12]	Dementia	Dementia	Dementia
DSM^a^ 5 [13]	Major neurocognitive disorder	Mild neurocognitive disorder	Major neurocognitive disorder
ICD 10^b^ [11]	MCI	Dementia	Dementia
Overall	Dementia	Dementia	Dementia
Consensus judgement	Dementia		
Aetiological subtype			
Alzheimer’s disease probable [6]	X		X
Alzheimer’s disease possible [6]		X	
Ischaemic cerebrovascular disease dementia probable [7]			
Ischaemic cerebrovascular disease dementia possible [7]			
Mixed aetiology [13]			
Parkinson’s [13]			
Lewy Body Dementia probable [8, 9, 13]			
Lewy Body Dementia possible [8, 9, 13]			
Tauopathy/Frontotemporal dementia [10]			
Other (describe) [13]			
Uncertain [13]			
Consensus judgement	Alzheimer’s disease probable [6]		

Notes: For the main analysis participant XX1 would be classed as having dementia

^a^Diagnostic and statistical manual of mental disorders/

^b^International classification of diseases

We will also investigate how the prevalence of MCI and dementia, and the accuracy of tests for diagnosis, varies with the three different definitions. We will then define subtypes of dementia according to standard definitions: Alzheimer’s disease [6], vascular [7], frontotemporal [10] and Lewy body [9].

#### Follow-up

We will follow consenting participants electronically using their GP records for up to seven years to determine whether those who do not have dementia at baseline develop it later on, and whether participants who are identified as having dementia by the study specialist assessment subsequently have their diagnosis refined. We will use follow-up data to analyse the diagnostic accuracy of tests for the diagnosis of dementia in the future (“delayed verification”) but the reference standard for the cross-sectional diagnosis of dementia will not take account of information that occurred more than six months subsequent to the research clinic.

### Statistical methods

#### Sample size

Table 4 shows the sample size required for a given lower 95 % confidence interval (LCI) based on a specificity of 95 % and a prevalence of dementia of 75 %. We used standard tables for the sample size calculation [75].

**Table 4:** Sample size for diagnostic test accuracy study, assuming specificity of 95 % and prevalence of dementia of 75 %

Lower 95 % confidence interval of specificity	Number of healthy people needed for lower confidence interval	Number of people with dementia needed for lower confidence interval	Total sample size
85 %	93	279	372
80 %	50	150	200
75 %	34	102	136

Other investigators have reported a specificity of between 89 % [29] and 94 % [76] for unaided “gut feeling” of GPs for diagnosing dementia, and individual tests such as clock draw (specificity 96 % [33]), Timed up-and-go (specificity 89 % [33]) and Phototest (specificity 89 % [52]) also have high specificity. We aim to recruit a sample of between 200-300 people. Using the five events per variable rule [77] this would allow us to evaluate between 30 and 45 diagnostic indicators (at 75 % prevalence of dementia).

#### Analysis

The plan for analysis may change with methodological advances in diagnostic science, but the current plan is outlined. We will construct 2x2 tables for each diagnostic indicator in the index battery and the outcome dementia, as defined by the consensus panel. We will also evaluate the discriminative ability of diagnostic indicators by calculating the area under the curve.

We will use logistic regression models with the target condition (dementia) as defined by the consensus panel being the binary dependent variable, and the diagnostic indicators as the independent variable. We will only include diagnostic indicators that have a p value of less than 0.10 in univariable logistic regression in the multivariable analysis. When we perform multivariable analysis we will include diagnostic indicators in the order in which they would be performed in clinical practice, for example age and sex (if significant), followed by GP “gut feeling” (if significant) followed by any tests in the index battery (ordered by mean average time taken to perform test). This will allow us to calculate the diagnostic accuracy of (e.g.) “gut feeling” allowing for the contribution of age and sex. We will calculate standard measures of diagnostic accuracy (sensitivity, specificity, likelihood ratios, area under the curve and predictive values) together with 95 % confidence intervals. We will use the regression coefficients to calculate predicted risks of dementia in participants and compare these to the actual risk using goodness of fit tests [78]. We will also consider analysing decision curves and evaluating the net reclassification index and integrated discrimination index [79].

Missing values will be imputed using multiple imputation by chained equations [80, 81]. We will perform a bootstrapping procedure to validate the final model and shrink the regression parameters [82]. We will use the model to construct a diagnostic algorithm and decision rule for use in clinical practice.

#### Qualitative evaluation of acceptability and feasibility

We will use joint interviews with a subsample of approximately 30 patients and their carers who attended a research clinic to determine how acceptability they would find a GP based diagnosis of dementia. The interview will be conducted after the research clinic. Participants will be purposefully sampled on the characteristics of age and GP practice (as a measure of deprivation and experience of general practice). We will not select people based on their diagnosis as this will not be known to the researcher at the research clinic. We will not offer participation when the researchers considers this would be burdensome for participants and their informants, and that this means that people with more severe cognitive impairment are unlikely to participate. Interviews will continue until saturation is reached. The topic guide will explore participants’ experience of seeing their GP about possible dementia, and then ask questions about the acceptability and perceived benefits and disadvantages of a GP based diagnosis of dementia. We will use focus groups with clinicians and managers in approximately five local general practices to identify the feasibility and barriers to a diagnostic evaluation for dementia taking place in general practice.

## Discussion

The TIMeLi study will be the first study, to our knowledge, to prospectively evaluate the diagnostic accuracy of a range of indicators in symptomatic people in primary care. The particular strengths of the study are the range of index tests that will be evaluated and the ability to account for the “gut feeling” of GPs. In addition, the study is being conducted in primary care with testing being delivered by a GP.

We anticipate our results will help address uncertainty about what tests are most useful to a GP to evaluate someone for possible dementia. If we identify a set of tests or diagnostic algorithm with high accuracy for diagnosing dementia then individual GPs could apply this in their clinical practice. Subsequent further work to evaluate this could lead to some people with established dementia being evaluated and diagnosed entirely in primary care, without specialist input. This does not preclude the use of neuroimaging, to help determine the likely aetiology of the dementia or to exclude alternative diagnoses, or the role of specialists for younger patients, more complex scenarios, or to provide aetiological diagnosis. Our results will inform the diagnostic approach to patients with possible dementia in primary care.

## Abbreviations

BASDEC, Brief Assessment Schedule Depression Cards; CRN, Clinical Research Network; CT, computed tomogram; DAT, dopamine transporter imaging; DSM, diagnostic and statistical manual of mental disorders; GP, general practitioner; ICD, International classification of diseases tenth edition; IQCODE, informant questionnaire for cognitive disorders in the elderly; LCI, lower confidence interval; M@T, memory alteration test; MMSE, mini mental state examination; MOCA, Montreal Cognitive Assessment; MRI, magnetic resonance imaging; NHS, National Health Service; NIHR, National Institute for Health Research; SPECT, single photon emission computed tomography; UK, United Kingdom; USA, United States of America
