# Erratum to: Towards improving diagnosis of memory loss in general practice: TIMeLi diagnostic test accuracy study protocol

**DOI:** 10.1186/s12875-016-0510-3

**Published:** 2016-08-27

**Authors:** Sam T. Creavin, Sarah J. Cullum, Judy Haworth, Lesley Wye, Antony Bayer, Mark Fish, Sarah Purdy, Yoav Ben-Shlomo

**Affiliations:** 1 School of Social and Community Medicine, University of Bristol, 39 Whatley Road, Bristol, BS8 2PS, UK; 2 North Bristol NHS Trust Southmead Hospital, Southmead Road, Westbury-on-Trym, Bristol, BS10 5NB UK; 3 Division of Population Medicine, Cardiff University School of Medicine University Hospital Llandough, Penarth, CF64 2XX, UK; 4 Department of Neurology, Musgrove Park Hospital, Taunton, Somerset, TA1 5DA, UK

## Erratum

Unfortunately, the original version of this article [1] mistakenly did not include an Acknowledgements section. The Acknowledgements have been included in full in this erratum.

## Acknowledgements

This paper presents independent research part-funded by the National Institute for Health Research School for Primary Care Research (NIHR SPCR). The views expressed are those of the author(s) and not necessarily those of the NIHR, the NHS or the Department of Health.
